# Icariin Attenuates Amyloid-β (Aβ)-Induced Neuronal Insulin Resistance Through PTEN Downregulation

**DOI:** 10.3389/fphar.2020.00880

**Published:** 2020-06-09

**Authors:** Xiaomei Zou, Xiyao Feng, Yalin Fu, Yuyang Zheng, Mingke Ma, Changhua Wang, Yemin Zhang

**Affiliations:** ^1^Neurology Center, The Second People’s Hospital of Jingzhou City, Jingzhou, China; ^2^2018 Clinical Medicine, Hubei University of Medicine, Shiyan, China; ^3^Department of Pathology & Pathophysiology, Wuhan University School of Basic Medical Sciences, Wuhan, China; ^4^Hubei Provincial Key Laboratory of Developmentally Originated Disease, Wuhan, China

**Keywords:** icariin, amyloid-β, insulin resistance, phosphatase and tensin homologue deleted on chromosome 10, neuronal cells

## Abstract

Neuronal insulin resistance is implicated in neurodegenerative diseases. Icariin has been reported to improve insulin resistance in skeletal muscle cells and to restore impaired hypothalamic insulin signaling in the rats with chronic unpredictable mild stress. In addition, icariin can exert the neuroprotective effects in the mouse models of neurodegenerative diseases. However, the molecular mechanisms by which icariin affects neuronal insulin resistance are poorly understood. In the present study, amyloid-β (Aβ) was used to induce insulin resistance in human neuroblastoma SK-N-MC cells. Insulin sensitivity was evaluated by measuring insulin-stimulated Akt T308 phosphorylation and glucose uptake. We found that the phosphatase and tensin homologue deleted on chromosome 10 (PTEN) mediated Aβ-induced insulin resistance. Icariin treatment markedly reduced Aβ-enhanced PTEN protein levels, leading to an improvement in Aβ-induced insulin resistance. Accordingly, PTEN overexpression obviously abolished the protective effects of icariin on Aβ-induced insulin resistance. Furthermore, icariin activated proteasome activity. The proteasome inhibitor MG132 attenuated the effects of icariin on PTEN protein levels. Taken together, these results suggest that icariin protects SK-N-MC cells against Aβ-induced insulin resistance by activating the proteasome-dependent degradation of PTEN. These findings provide an experimental background for the identification of novel molecular targets of icariin, which may help in the development of alternative therapeutic approaches for neurodegenerative diseases.

## Introduction

Insulin influences energy metabolism and growth, which are mostly mediated by the activation of the PI3K/Akt pathway and the Ras/MAPK pathway, respectively. The brain is one of the major targets of insulin. Through binding with its specific receptor and activating its downstream signaling, insulin exhibits central neuromodulatory functions. For example, insulin has been reported to be involved in the regulation of synaptic formation, learning and memory, cognition, neuronal stem cell activation, and neuronal apoptosis (Tumminia et al., 2018). Therefore, insulin deficiency or impairment of insulin signaling (namely, insulin resistance) in the central nervous system (CNS) can result in brain disorders.

In the past decades, growing evidence from clinical and laboratory studies has linked insulin resistance to neurodegenerative disorders, such as Parkinson’s disease (PD) and Alzheimer’s disease (AD) (Schubert et al., 2004; Athauda and Foltynie, 2016; Tumminia et al., 2018). Insulin resistance can indeed affect tau phosphorylation, the metabolism of the amyloid precursor protein, and clearance of beta-amyloid (Aβ) from the brain, thus contributing to the development of neurodegenerative diseases (Plum et al., 2005).

Icariin, a major bioactive component extracted from plants of the *Epimedium* genus (Liu et al., 2006), has been shown to ameliorate insulin resistance in skeletal muscle cells (Han et al., 2015; Li et al., 2018; Liu et al., 2019) and restore impaired hypothalamic insulin signaling in the rats with chronic unpredictable mild stress (Pan et al., 2013). Currently, icariin has been found to evoke the neuroprotective effects *in vivo*. Indeed, icariin may improve learning and memory dysfunction, as well as synaptic and cognitive deficits, in the AD mice (Urano and Tohda, 2010; Chen et al., 2016; Sheng et al., 2017). In the PD mice, icariin has also been reported to reduce dopaminergic neuronal loss and neuroinflammation (Wang et al., 2018; Zhang et al., 2019). However, the molecular mechanisms underlying the effects of icariin on neuronal insulin resistance remain elusive, although the neuroprotective properties of icariin in PD mice were reported to be associated with its regulation of the PI3K/Akt and the MEK/ERK signaling pathways (Chen et al., 2017).

In the present study, we found that icariin significantly attenuated Aβ-induced insulin resistance in human neuroblastoma SK-N-MC cells. We also confirmed that the enhancement of proteasome activity by icariin treatment contributed to the degradation of the phosphatase and tensin homologue deleted on chromosome 10 (PTEN). These findings provide an experimental background for the detection of novel molecular targets of icariin, which may be useful for the development of alternative therapeutic approaches for neurodegenerative conditions.

## Methods

### Cell Culture and Treatment

Human neuroblastoma SK-N-MC cells (ATCC, Bethesda, MD, USA) were maintained in Minimum Essential Media (MEM, GIBCO-BRL, Gaithersburg, MD, USA) supplemented with 10% fetal bovine serum (FBS), 100 units/ml penicillin, 100 μg/ml streptomycin, and 2 mM L-glutamine at 37°C and 5% CO_2_. The cells were treated with 2.5 mM of Aβ 1-42 for 24 h, to induce insulin resistance (Kornelius et al., 2017). Aβ 1-42 was acquired from AnaSpec Inc. (San Jose, CA, USA), dissolved in 100% 1,1,1,3,3,3-hexafluoro-2-propanol, and then dried using a vacuum desiccator. Next, Aβ was resuspended in dimethylsulfoxide (DMSO) and stored at -20°C (Kornelius et al., 2017). Icariin (I1286) was obtained from Sigma-Aldrich, Corp. (St. Louis, MO, USA) and dissolved in DMSO. The final concentration of DMSO in the cell supernatant was 1% (v/v). To measure insulin signaling and glucose uptake, the cells were serum-starved for 6 h and then stimulated with 100 nM of insulin for 10 min.

### Small Interfering RNA (siRNA) and Transfection

PTEN siRNA (#6538) and control siRNA (#6201) were obtained from Cell Signaling Technology (Beverly, MA, USA). Transfection of siRNAs was performed according to the manufacturer’s protocol. Western blot analysis was performed to measure the transfection efficiency.

### Quantitative Real-Time RT-PCR (RT-qPCR)

RT-qPCR was performed as described previously (Li et al., 2018). The primer sequences used were as follows: PTEN forward, 5′-GTCACTGCTTGTTGTTTGC-3′ and reverse, 5′-TTCTTTGTTGATAGCCTCCAC-3′; U6 forward, 5′-GCTTCGGCAGCACATATACTAAAAT-3′ and reverse, 5′-CGCTTCACGAATTTGCGTGTCAT-3′. Relative gene expression was quantified by the 2^-ΔΔCt^ method and normalized to U6. All samples were analyzed in triplicates.

### Adenovirus/PTEN Construction and Transduction

Adenovirus carrying the human PTEN gene (Ad/PTEN) was constructed as described previously (Stewart et al., 2002). Adenoviruses encoding β-galactosidase (Ad/β-gal) were kindly provided by Dr. Xuejun Wang (The University of South Dakota Sanford School of Medicine, SD, USA). The cells were incubated in serum-free medium containing adenoviruses for 6 h, and then grown in growth medium for another 36 h. Forty-two hours after virus infection, the cells were pending for experimental study. Ad/β-gal served as a negative control. The optimal multiplicity of infection (MOI) used in the experiments was 50 (Zhang et al., 2015).

### Proteasome Peptidase Activity Assays

The chymotrypsin-like activity of the 20S proteasome in the cells was measured as described previously (Zhang et al., 2015; Li et al., 2018). Briefly, the cells were lysed with cytosolic extraction buffer (50 mM HEPES, pH 7.5, 20 mM KCl, 5 mM MgCl_2_, 2 mM ATP, 1 mM DTT, 0.025% Digitonin). The lysate was centrifuged at 10,000 × *g* for 10 min at 4°C, and the supernatants were collected. Protein concentration was determined through the bicinchoninic acid (BCA) assay. The synthetic fluorogenic peptide substrate Suc-LLVY-Amc (Boston Biochem, Cambridge, MA, USA) was used for assaying chymotrypsin-like peptidase activity. For assay specificity, 1 μM of proteasome inhibitor MG132 (S2619, Selleck, Shanghai, CN) was incubated with the extract. After 90 min of incubation at 37°C, the fluorescence intensity was read (excitation, 350 nm; emission, 440 nm) using fluorescence spectrometer (Perkin Elmer precisely LS 55, Billerica, MA, USA).

### Glucose Uptake Assay

The Glucose Uptake Assay Kit (Colorimetric) (ab136955, Abcam Inc., Cambridge, MA, USA) was used to measure glucose uptake according to the manufacturer’s protocol.

### Western Blot Analysis

Quantification of proteins or phosphorylated proteins was performed by western blotting, as described previously (Zhang et al., 2015; Li et al., 2018). Briefly, the cells were lysed in lysis buffer (50 mM HEPES, pH 7.6, 150 mM NaCl, 1% Triton X-100, 10 mM NaF, 20 mM sodium pyrophosphate, 20 mM β-glycerol phosphate, 1 mM sodium orthovanadate, 10 μg/ml leupeptin, 10 μg/ml aprotinin, and 1 mM phenylmethanesulfonyl fluoride). The lysates were incubated on ice for 20 min and then centrifuged at 14,000 × *g* for 10 min at 4°C. The supernatants were mixed with an equal volume of 2 x SDS-PAGE loading buffer and then heated at 95°C for 10 min. The proteins were separated by SDS-PAGE, transferred to a nitrocellulose membrane, and detected with specific antibodies.

### Statistical Analysis

The data are presented as the mean ± standard deviation (SD). The differences between the groups were examined for statistical significance using analysis of variance (ANOVA), followed by the Newman–Keuls *post hoc* test. A *p*-value <0.05 was considered statistically significant. The figures are the representative of at least four independent experiments with similar results.

## Results

### Icariin Improved Aβ-Induced Neuronal Insulin Resistance

**Figure 1:**
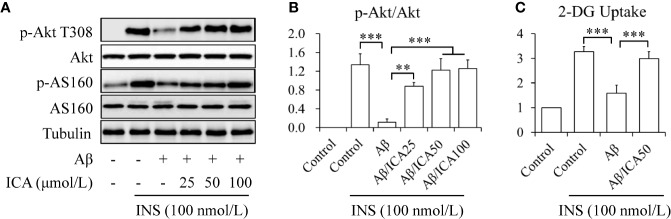
Icariin mitigated Aβ-induced insulin resistance in SK-N-MC cells. **(A)** Effect of icariin (ICA) on insulin (INS)-stimulated phosphorylation of Akt T308 and AS160. **(B)** Quantification of p-Akt T308 shown in **(A)**. **(C)** Effect of ICA on INS-stimulated 2-deoxy-D-glucose (2-DG) uptake. N = 4. **p < 0.01, ***p < 0.001 *vs.* indicated group.

To observe the potential effects of icariin on neuronal insulin resistance, human neuroblastoma SK-N-MC cells were serum-starved for 6 h and then treated with or without 2.5 mM of Aβ 1-42, in the presence or absence of 25, 50, or 100 μM of icariin for 24 h, followed by stimulation with or without 100 nM of insulin for 10 min. Aβ was used to induce neuronal insulin resistance *in vitro* (Zhao et al., 2008; Najem et al., 2016; Sajan et al., 2016; Wani et al., 2019) and insulin was administered to stimulate insulin signaling. As shown in **Figure 1**, icariin treatment reversed the inhibitory effects of Aβ on insulin-stimulated phosphorylation of Akt at Thr308 and of its downstream substrate AS160 (**Figures 1A, B**). To test the effects of icariin and Aβ on glucose uptake, SK-N-MC cells were serum-starved for 6 h and then treated with or without 2.5 mM of Aβ 1-42, in the presence or absence of 50 μM of icariin for 24 h, followed by stimulation with or without 100 nM of insulin for 10 min. We found that icariin treatment significantly increased 2-deoxy-D-glucose (2-DG) uptake (**Figure 1C**) under stimulation with insulin, when compared with Aβ treatment alone. These results suggested that icariin treatment protected SK-N-MC cells against Aβ-induced insulin resistance.

### PTEN Was Involved in Aβ-Induced Insulin Resistance

**Figure 2:**
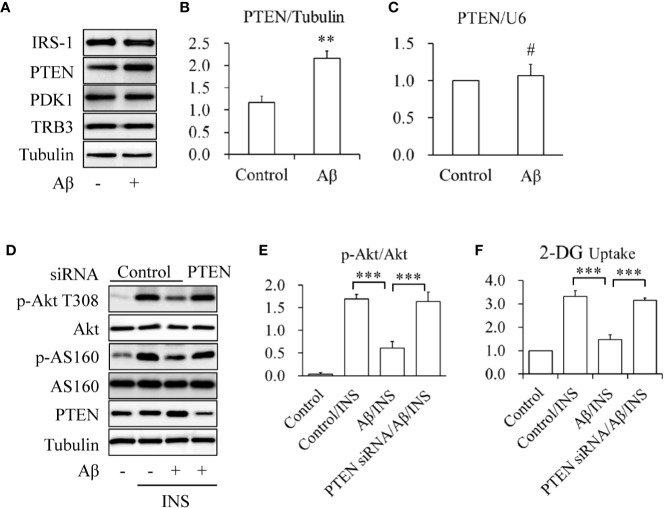
Aβ elevated PTEN protein levels in SK-N-MC cells. **(A, B)** Effect of Aβ on PTEN protein levels. **(C)** Effect of Aβ on PTEN mRNA levels. **(D)** Effect of PTEN knockdown on Aβ-induced insulin resistance. **(E)** Quantification of p-Akt T308 shown in **(D)**. **(F)** Effect of PTEN knockdown on INS-stimulated 2-deoxy-D-glucose (2-DG) uptake in Aβ-treated cells. N = 4. **p < 0.01, ***p < 0.001 *vs.* indicated group; ^#^*p* > 0.05 *vs.* control group.

To determine the mechanisms underlying the protective effects of icariin on Aβ-induced insulin resistance, the expression levels of key proteins involved in the regulation of Akt phosphorylation, such as IRS-1, PTEN, PDK1, and TRB3, were investigated. SK-N-MC cells were serum-starved for 6 h and then treated with or without 2.5 mM of Aβ 1-42 for 24 h. We found that Aβ 1-42 treatment significantly enhanced only PTEN protein levels (**Figures 2A, B**) whereas PTEN mRNA levels remained unchanged (**Figure 2C**). To confirm the role of PTEN in Aβ-induced insulin resistance, PTEN was knocked down (KD) in SK-N-MC cells by siRNA techniques. Wild-type or PTEN KD cells were treated with or without 2.5 mM of Aβ 1-42 for 24 h, followed by stimulation with or without 100 nM of insulin for 10 min. We found that PTEN depletion markedly prevented Aβ-induced reduction of insulin-stimulated Akt T308 phosphorylation (**Figures 2D, E**) and glucose uptake (**Figure 2F**). These results suggested that the increased PTEN levels were responsible for Aβ-induced neuronal insulin resistance *in vitro*.

### Icariin-Induced PTEN Degradation Contributed to Its Protective Effects Against Aβ-Induced Insulin Resistance

**Figure 3:**
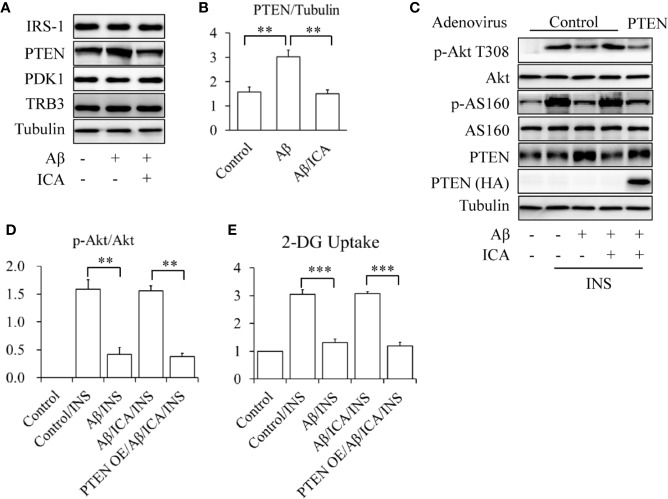
Icariin reduced the Aβ-induced elevated PTEN protein levels in SK-N-MC cells. **(A, B)** Effect of icariin (ICA) on PTEN protein levels. **(C)** Overexpression of PTEN (PTEN OE) abolished the beneficial effects of ICA on insulin signaling. **(D)** Quantification of p-Akt T308 shown in **(C)**. **(E)** Effect of PTEN OE on INS-stimulated 2-deoxy-D-glucose (2-DG) uptake in the cells co-treated with Aβ and ICA. N = 4. **p < 0.01, ***p < 0.001 *vs.* indicated group.

To check the effects of icariin on PTEN, SK-N-MC cells were serum-starved for 6 h and then treated with or without 2.5 mM of Aβ 1-42, in the presence or absence of 50 μM of icariin for 24 h. We found that icariin treatment significantly reduced Aβ-enhanced PTEN protein levels (**Figures 3A, B**). To investigate whether PTEN is a target of icariin’s activity, a PTEN rescue experiment was performed, in which PTEN was overexpressed by infecting the cells with Ad/PTEN. Ad/β-gal acted as a negative control. The control or PTEN-overexpressing SK-N-MC cells were serum-starved for 6 h and then treated with or without 2.5 mM of Aβ 1-42, in the presence or absence of 50 μM of icariin for 24 h, followed by stimulation with 100 nM of insulin for 10 min. We found that the PTEN-overexpressing SK-N-MC cells displayed a reduction in insulin-stimulated Akt T308 phosphorylation (**Figures 3C, D**) and glucose uptake (**Figure 3E**) in response to the combined treatment with Aβ and icariin, when compared with the control cells.

**Figure 4:**
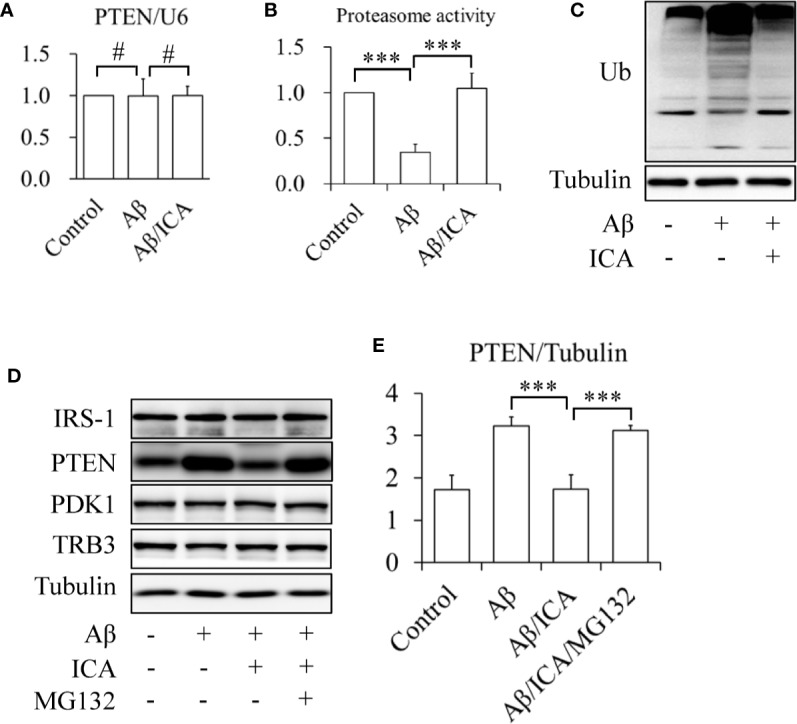
Icariin activated proteasome leading to the degradation of PTEN in SK-N-MC cells. **(A)** Effect of icariin (ICA) on PTEN mRNA levels. **(B)** Effect of ICA on the proteasome activity. **(C)** Effect of ICA on the ubiquitination of total proteins. **(D, E)** The proteasome inhibitor MG132 abolished the effect of ICA on PTEN protein levels. N = 4. ***p < 0.001 *vs.* indicated group; ^#^*p* > 0.05 *vs.* indicated group.

In addition, the results of the RT-qPCR revealed that icariin did not affect the mRNA levels of PTEN (**Figure 4A**, **Figure S1**). Interestingly, Aβ treatment significantly inhibited proteasome activity, which was prevented by icariin administration (**Figure 4B**). In line with this finding, we also observed that icariin administration reduced the Aβ-enhanced ubiquitination of total proteins (**Figure 4C**). These findings suggested that icariin reduced PTEN protein levels *via* a proteasome-dependent mechanism. To confirm this hypothesis, SK-N-MC cells were serum-starved for 6 h, pretreated with 2 μM of proteasome inhibitor MG132 for 30 min, and then treated with or without 2.5 mM of Aβ 1-42, in the presence or absence of 50 μM of icariin for 24 h. We found that MG132 administration significantly increased PTEN protein levels, when compared with the group that received a combined treatment of Aβ and icariin (**Figures 4D, E**).

Taken together, these results suggested that icariin activated the proteasome, leading to a reduction of PTEN protein levels, by which icariin exhibited its protective effects against Aβ-induced neuronal insulin resistance.

## Discussion

Previous studies have demonstrated the interactions between insulin and Aβ. Insulin can inhibit Aβ aggregation, enhance Aβ degradation, and prevent Aβ-induced cellular events, such as membrane disruption and tau phosphorylation (Yamamoto et al., 2018; Long et al., 2019). On the other hand, Aβ has also been found to modulate insulin signaling. For example, Aβ can reduce the responsiveness to insulin in presynaptic terminals (Heras-Sandoval et al., 2012), decrease insulin expression in cultured astrocytes (Takano et al., 2018), impair neuronal insulin receptors (Zhao et al., 2008), and induce insulin resistance in neuronal cells and peripheral cells (Zhang et al., 2012; Najem et al., 2016; Wani et al., 2019). These deleterious effects of Aβ on insulin action or insulin sensitivity may be mediated by activating the JAK2/STAT3/SOCS-1 or JNK signaling pathways (Zhang et al., 2012; Najem et al., 2016).

In the present study, we found that Aβ increased PTEN protein levels (**Figures 2A, B**), which is consistent with previous work showing the involvement of PTEN proteins in the Aβ-induced toxicity observed in organotypic hippocampal slice cultures (Nassif et al., 2007). The tumor suppressor PTEN is a phosphoinositide 3-phosphatase that serves as the main negative regulator of class I phosphoinositide-3-kinases (PI3Ks) (Leslie and Downes, 2002). Therefore, Akt can be indirectly dephosphorylated and inactivated by PTEN. In line with this, we found that Aβ-reduced insulin-stimulated Akt phosphorylation and glucose uptake were significantly mitigated by PTEN depletion (**Figures 2A–F**). Given that the mRNA levels of PTEN remained unchanged by Aβ treatment (**Figure 2C**), these findings suggested that Aβ induced neuronal insulin resistance by upregulating PTEN protein levels.

In addition, we found that icariin treatment significantly suppressed Aβ-enhanced PTEN protein levels (**Figures 3A, B**), but had no effect on PTEN mRNA levels (**Figure 4A**, **Figure S1**). The improvement of insulin-stimulated Akt phosphorylation and glucose uptake by icariin treatment was also abolished by PTEN overexpression (**Figures 3C–E**). These findings indicated that the protective effect of icariin against Aβ-induced insulin resistance was mediated by PTEN.

Some miRNAs have been shown to modulate PTEN expression at the post-transcriptional level. For example, both of miR-186 and miR-21 can negatively regulate gene expression by binding to the 3′-untranslated region (3′-UTR) of PTEN mRNAs, leading to mRNA degradation or translational repression (Bermúdez Brito et al., 2015; Feng et al., 2019). Interestingly, icariin can upregulate these miRNAs (Lian et al., 2018; Ma et al., 2018), suggesting a possibility of that miR-186 or miR-21 may mediate the inhibitory effects of icariin on PTEN. However, our RT-qPCR results revealed that neither Aβ nor icariin affected the expression levels of miR-186 and miR-21 in SK-N-MC cells (data not shown). In addition, our results showed that PTEN mRNA levels did not significantly change following treatment with Aβ or icariin (**Figures 2C** and **4A**, **Figure S1**). These findings indicated that the inhibitory effects of icariin on PTEN were independent of miRNAs.

It has been demonstrated that the ubiquitin-proteasome system plays a critical role in the degradation of PTEN protein (Wu et al., 2003). One reason for the increased PTEN protein levels induced by Aβ may be its inhibitory effect on proteasome activity (Tseng et al., 2008). Indeed, we found that Aβ treatment significantly reduced proteasome activity in SK-N-MC cells (**Figure 4B**). Icariin has been proven to activate the proteasome (Zhu et al., 2011; Li et al., 2018). As an inhibitor of phosphodiesterase type 5 (PDE5) (Xin et al., 2003; Rahimi et al., 2010), icariin indirectly stimulates proteasome activity by activating the cGMP/PKG pathway (Ranek et al., 2013). In addition, icariin can also directly increase the expression levels of some proteasome subunits, such as proteasome subunit-alpha type 6 and type 2, and reverse the inhibitory effects of the proteasome inhibitor epoxomicin on proteasome activity (Zhu et al., 2011). In the present study, we found that icariin administration significantly protected SK-N-MC cells against Aβ-suppressed proteasome activity, and reduced total protein ubiquitination (**Figures 4B, C**). Furthermore, the inhibition of proteasome activity by its specific inhibitor MG132 abolished the effects of icariin on PTEN in Aβ-treated cells (**Figures 4D, E**). These findings suggested that icariin activated the proteasome, leading to PTEN degradation.

In summary, icariin exposure improved Aβ-induced neuronal insulin resistance, which is mediated by a proteasome-associated PTEN degradation. These findings suggest that icariin might be a novel therapeutic agent for the treatment of neurodegenerative diseases, such as the PD and the AD. However, further studies are needed to elucidate the mechanisms by which icariin regulates insulin signaling in the CNS *in vivo*.

### Limitation

In the present study, we described the molecular mechanisms by which icariin mitigated Aβ-induced neuronal insulin resistance. However, the majority of the results were based on Aβ-treated SK-N-MC cells, which represent an *in vitro* model of uncertain relevance to AD. In addition, the insulin concentration used in the performed experiments was higher than the physiological one. The concentrations of icariin were also relatively high. Therefore, further studies are necessary to obtain more potent derivatives of icariin, as well as to provide further *in vivo* evidence in experimental settings closer to clinical reality.

## Data Availability Statement

The datasets generated for this study are available on request to the corresponding author.

## Author Contributions

YeZ and CW designed the study. XZ, XF, YF, YuZ, MM, and YeZ performed the experiments. XZ, YeZ, and CW conducted the statistical analyses. YeZ wrote the manuscript. All authors approved submission of the manuscript.

## Funding

This work was supported by the National Natural Science Foundation of China (Grant No. 81870550) and the Medical Science Advancement Program (Basic Medical Sciences) of Wuhan University (Grant No. TFJC2018001).

## Conflict of Interest

The authors declare that the research was conducted in the absence of any commercial or financial relationships that could be construed as a potential conflict of interest.
