# Supplementary material for: Icariin Attenuates Amyloid-β (Aβ)-Induced Neuronal Insulin Resistance Through PTEN Downregulation
Source: Front Pharmacol. 2020 Jun 9;11:880. doi: 10.3389/fphar.2020.00880 (PMC7296100; doi:10.3389/fphar.2020.00880)
Supplement: Supplementary file 1 [file DataSheet_1.pdf]

SUPPLEMENTARY MATERIAL

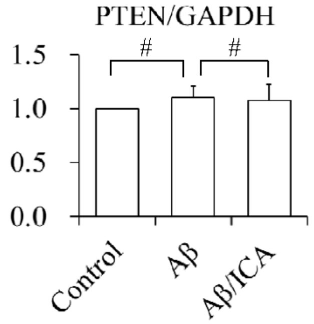

FIGURE S1. The effect of Aβ and icariin on PTEN mRNA levels. SK-N-MC cells were serum-starved for 6 h and then treated with or without 2.5 mM of Aβ 1-42, in the presence or absence of 50 μM of icariin for 24 h. RT-qPCR was performed as described in Methods. GAPDH was used as an internal control. The primer sequences of human GAPDH (NM_002046.4) was as follows: forward 5'-GTCTCCTCTGACTTCAACAGCG-3', reverse 5'-ACCACCCTGTTGCTGTAGCCAA-3'. N = 4. # $p > 0.05$ *vs*. indicated group.
